# Quinoline Compounds Targeting the *c*-Ring of ATP Synthase Inhibit Drug-Resistant *Pseudomonas aeruginosa*

**DOI:** 10.1021/acsinfecdis.3c00317

**Published:** 2023-11-03

**Authors:** Vesper M. Fraunfelter, Bryce A. Pugh, Alexander P. L. Williams, Katie T. Ward, Dietrich O. Jackson, Molly Austin, John F. Ciprich, Lorelei Dippy, Jason Dunford, G. Nathaniel Edwards, Evan Glass, Kyle M. Handy, Casey N. Kellogg, Kaitlyn Llewellyn, K. Quinn Nyberg, Sam J. Shepard, Casey Thomas, Amanda L. Wolfe, P. Ryan Steed

**Affiliations:** Department of Chemistry and Biochemistry, University of North Carolina Asheville, One University Heights, Asheville, North Carolina 28804, United States

**Keywords:** ATP synthase, bioenergetics, antibiotics, antibiotic resistance, quinolines, Pseudomonas aeruginosa

## Abstract

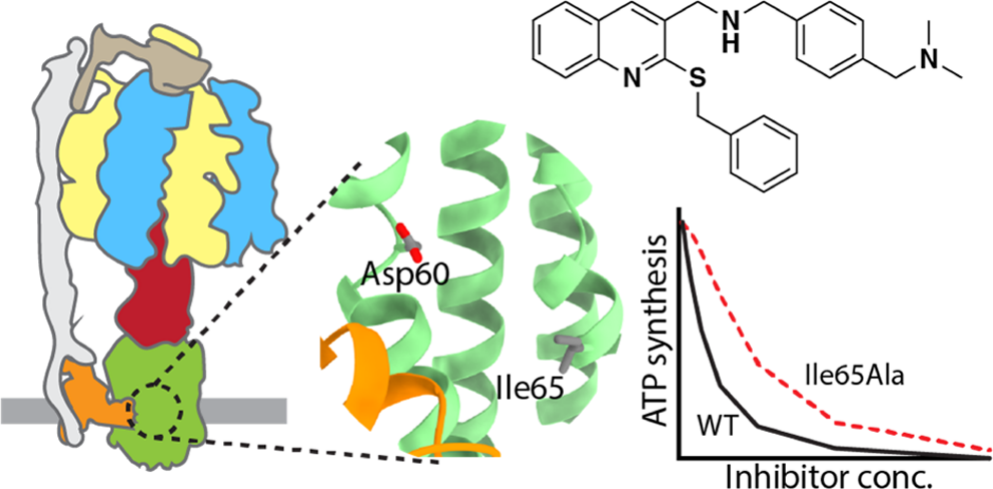

*Pseudomonas aeruginosa* (PA) is a Gram-negative, biofilm-forming bacterium and an opportunistic pathogen. The growing drug resistance of PA is a serious threat that necessitates the discovery of novel antibiotics, ideally with previously underexplored mechanisms of action. Due to their central role in cell metabolism, bacterial bioenergetic processes are of increasing interest as drug targets, especially with the success of the ATP synthase inhibitor bedaquiline to treat drug-resistant tuberculosis. Like *Mycobacterium tuberculosis*, PA requires F_1_F_o_ ATP synthase for growth, even under anaerobic conditions, making the PA ATP synthase an ideal drug target for the treatment of drug-resistant infection. In previous work, we conducted an initial screen for quinoline compounds that inhibit ATP synthesis activity in PA. In the present study, we report additional quinoline derivatives, including one with increased potency against PA ATP synthase *in vitro* and antibacterial activity against drug-resistant PA. Moreover, by expressing the PA ATP synthase in *Escherichia coli*, we show that mutations in the H^+^ binding site on the membrane-embedded rotor ring alter inhibition by the reported quinoline compounds. Identification of a potent inhibitor and its probable binding site on ATP synthase enables further development of promising quinoline derivatives into a viable treatment for drug-resistant PA infection.

Multidrug-resistant (MDR) *Pseudomonas aeruginosa* (PA) is a Gram-negative, biofilm-forming bacterium that is prevalent in hospital settings and is especially dangerous for patients with chronic lung disease, such as cystic fibrosis, or those with weakened immune systems.^1,2^ MDRPA, which has been designated as a “serious threat” by the Center for Disease Control in their 2019 report, causes approximately 32,600 infections and 2700 deaths in the US per year and costs an estimated $767 million per year in healthcare-related expenses.^1^ Current antibiotics used to treat PA infections include β-lactams, aminoglycosides, cephalosporins, fluoroquinolones, and polymyxins.^3^ However, the majority of these treatments are ineffective against MDRPA due to intrinsically encoded or genetically acquired resistance for the most common antibacterial mechanisms of action (MOA) (i.e., cell wall synthesis inhibition/disruption, protein synthesis inhibition, and DNA/RNA synthesis inhibition).^3,4^ Additional resistance mechanisms specific to MDRPA are a tightly packed, polyanionic lipopolysaccharide outer membrane with reduced porin expression, which together prevent antibiotics from entering the cell, and upregulation of RND superfamily efflux pumps, including MexAB-OprM, MexXY-OprM, and MexCD-OprJ, which are involved in carbapenem, fluoroquinolone, and aminoglycoside resistance.^5−7^ Finally, MDRPA can also be less susceptible to antibiotics due to biofilm formation, which further prevents drug penetration.^4,7^

To circumvent existing resistance mechanisms in pathogenic bacteria, novel MOAs are needed that target essential enzymes. Bacterial bioenergetics processes are promising targets, not only because of their essential nature but also because bioenergetic insults can potentiate the activity of existing drugs.^8^ F_1_F_o_ ATP synthase catalyzes the final step in oxidative phosphorylation, which is a critical energy-producing pathway in all cells. While some bacterial species, e.g., *Escherichia coli* (EC), can circumvent ATP synthase during ATP synthesis via fermentation, *Pseudomonas* species rely on ATP synthase even during fermentative processes,^9^ making ATP synthase an ideal target for antibiotics. ATP synthase (Figure 1A) is a complex of rotary motors that (1) use the proton electrochemical gradient to generate rotation (F_o_) and (2) use the energy of rotation to synthesize ATP from ADP and phosphate (F_1_).^10^ In many species of bacteria, the motors can operate in reverse under certain conditions, hydrolyzing ATP to pump H^+^ into the periplasm to maintain a proton motive force. The bacterial F_o_ motor is composed of a rotor of 10–15 *c* subunits adjacent to subunit *a* and a dimer of *b* subunits that form the stator. Subunit *a* provides two aqueous half channels by which protons are shuttled to and from the H^+^-binding acidic residue (Asp60 in PA) on each *c* subunit (Figure 1B). The validity of F_o_ as a target for antibiotics has been established by the success of bedaquiline (BDQ) in the treatment of tuberculosis.^11^ In *Mycobacterium tuberculosis* (MT), BDQ specifically binds to the H^+^-binding sites on *c* subunits flanking the rotor–stator interface, resulting in inhibition of ATP synthesis and cell death.^12^ Macrolide inhibitors, including oligomycin and venturicidin, have also been shown to bind to the *c*-ring near H^+^-binding sites.^13−15^

**Figure 1:**
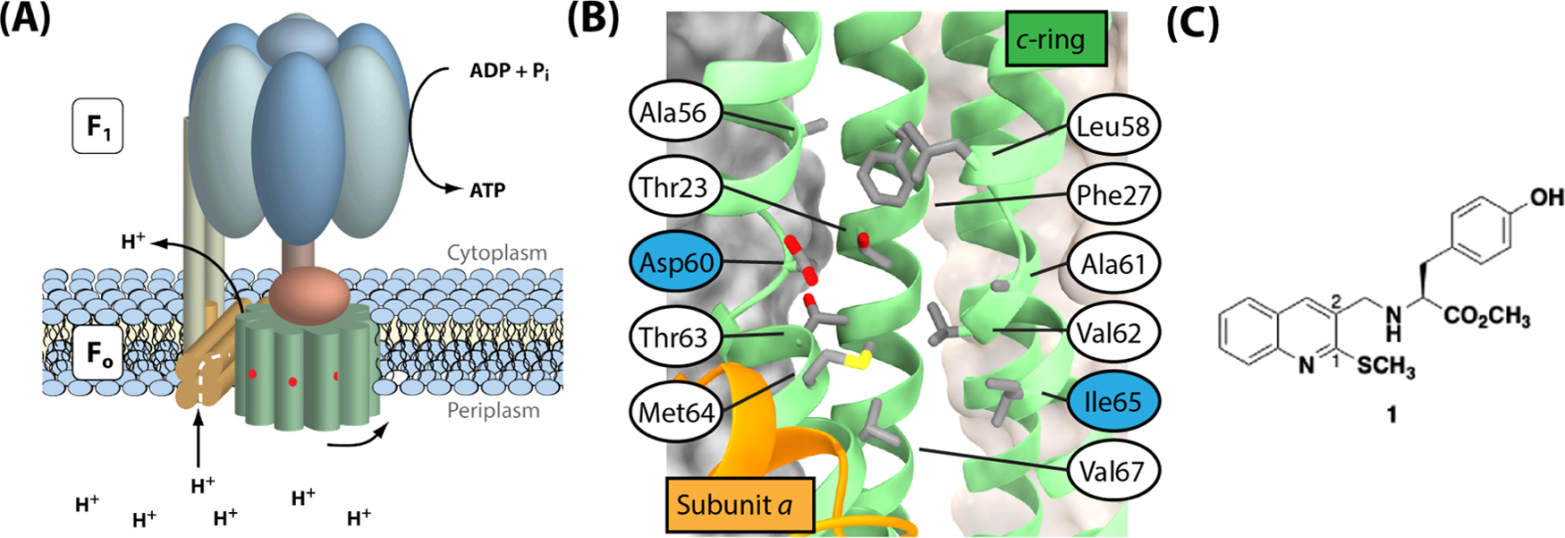
(A) Cartoon of ATP synthase showing the arrangement of F_1_F_o_ subunits in the bacterial inner membrane. (B) Model of the H^+^ binding site at the interface between two c subunits. The homology model was generated by SWISS MODEL^16^ based on the cryoelectron microscopy structure of ATP synthase from *Acinetobacter baumannii*,^17^ which is 77% identical and 92% similar in this region. (C) Compound **1** was previously reported as an inhibitor of PA ATP synthesis activity.^18^

Previously, we synthesized a series of C1 and C2 quinoline analogs in order to determine if ATP synthesis inhibition could be a useful antibiotic development target in PA.^18^ From this study, we found that 6 of the quinolines were able to inhibit ATP synthesis in PA vesicles, with compound **1** (Figure 1C), which has a methyl sulfide at C1 and a l-tyrosine methyl ester at C2, being among the most active of the compounds surveyed. Through this SAR study, we determined that quinolines with a C1 methyl sulfide and a bulky, hydrophobic group with the ability to hydrogen bond at C2 showed the greatest inhibitory effects. However, despite their ATP synthase inhibitory activity, none of the compounds were able to act as antibiotics against EC or PA in liquid culture due to poor accumulation in the cell. In this study, we report the synthesis and evaluation of new quinoline-derived compounds that inhibit PA ATP synthase and measurably inhibit the growth of drug-resistant PA. Furthermore, we show via mutagenesis that the novel quinoline compounds most likely bind to ATP synthase at the H^+^ binding site on the *c*-ring.

## Results and Discussion

### Synthesis and Initial Activity Screen

**Scheme 1:**
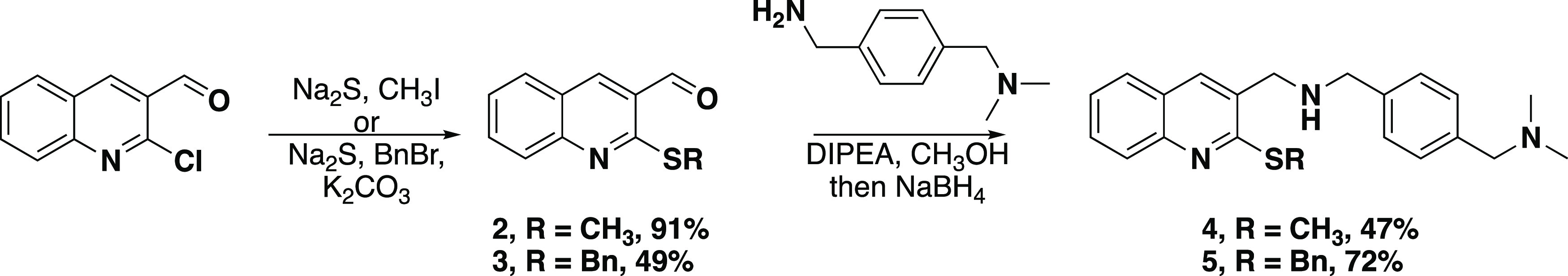
Synthesis of Compounds **4** and **5**

As a follow-up to our initial SAR study,^18^ a second series of quinoline derivatives (compounds **4**, **5**, **S1**–**6**) were synthesized, and a preliminary screen was conducted for antibiotic activity against PA. Compounds **4** and **5**, which contain a dimethylamine at C2, similar to BDQ, were synthesized via reductive amination of the methyl sulfide (**2**)^18^ or benzyl sulfide (**3**) quinoline-carbaldehyde with 4-dimethylaminomethylbenzylamine, respectively, in good yields (Scheme 1). Compounds **S1**–**S6**, which contain various aromatic functional groups at the C2 position, were synthesized via reductive amination (**S1**–**S5**) or condensation (**S6**) in moderate to low yields (Schemes S1/S2). In the initial antibacterial activity screen against a susceptible laboratory strain of EC (designated EC 25922), a nonvirulent, biofilm-forming strain of PA (designated PA 9027), and the PΔ6 efflux knockout strain of PA,^6^ compounds **4** and **5** were active against both EC and/or PΔ6 at a concentration of 128 μg/mL (Table S1). These two compounds were therefore evaluated further for antibacterial and ATP synthase inhibition activity.

### ATP Synthesis Inhibition and Antibacterial Activity of **4** and **5**

**Figure 2:**
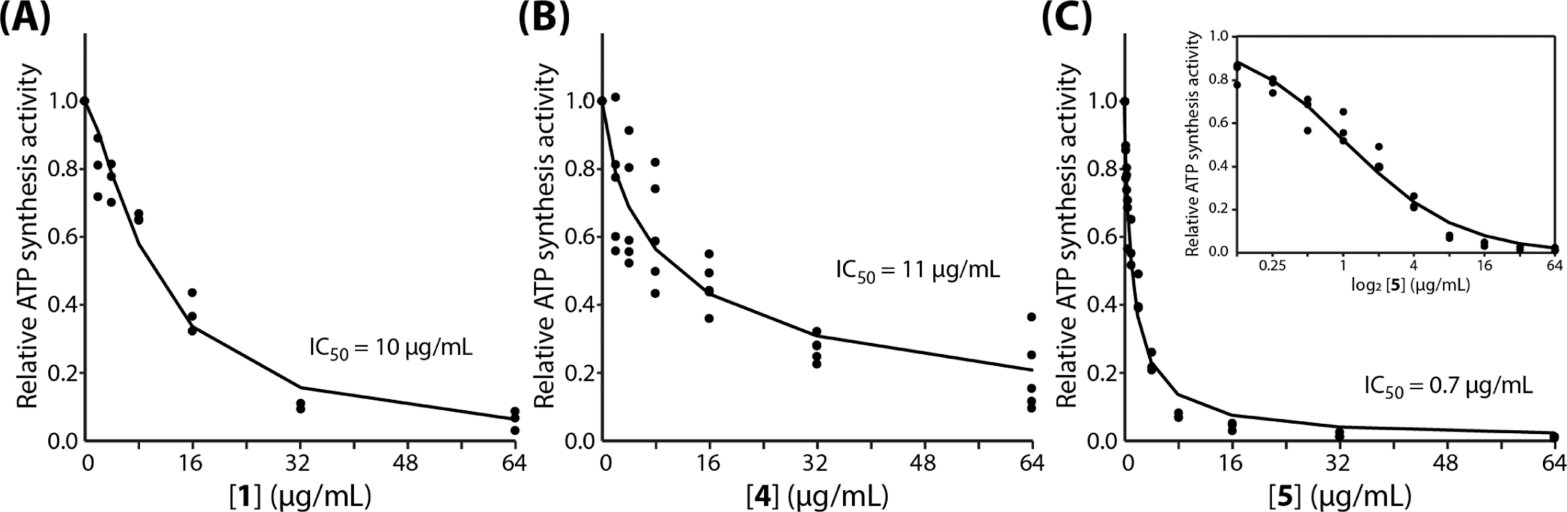
Inhibition of ATP synthesis activity in PA membrane vesicles. NADH-driven ATP synthesis activity of inverted membrane vesicles from PA was measured in the presence of 0–64 μg/mL compound **1** (A), **4** (B), or **5** (C) using an end point luminescence assay as described in Methods section. Luminescence values in each replicate (dots) were normalized to 0 μg/mL compound, and mean values were used to fit a dose–response curve (black line). Panel (C) inset shows the quality of fit at lower concentrations of compound **5** on a logarithmic (base 2) plot.

Since compounds **4** and **5** showed the most promising antibacterial activity in the initial screen, we first evaluated them for their ability to inhibit *in vitro* ATP synthesis activity in PA membrane vesicles. Inverted membrane vesicles were prepared from PA 9027 and evaluated for NADH-driven ATP synthesis activity using an end point luciferin/luciferase assay at increasing concentrations of compounds **4** and **5** (Figure 2). IC_50_ values calculated using a simple dose–response model are reported in Table 1. Compared to tyrosine quinoline **1**, which was one of the best PA ATP synthase inhibitors in our first SAR study with an IC_50_ = 10.0 μg/mL,^18^ compound **4** with the less bulky methyl sulfide at the C1 position was slightly less active with an IC_50_ = 11.1 μg/mL. However, compound **5** with the benzyl sulfide at the C1 position showed increased activity with an IC_50_ = 0.7 μg/mL. Since the putative binding site on the *c* subunit of PA ATP synthase is less sterically hindered and more hydrophobic than that same site in MT ATP synthase due to the changes of Asp (MT) to Phe27, Tyr (MT) to Thr63, Phe (MT) to Met64, and Leu(MT) to Val67 (Figure 1B), it is likely that the dimethylaminomethylbenzylamine substituent at the C2 position of both **4** and **5** is not large enough to fill this site, and therefore, the addition of the benzene at the C1 position of compound **5** increased binding.

**Table 1:** Inhibition of ATP Synthesis in PA and Antibacterial Activity of **4** and **5**

	antibacterial activity (MIC μg/mL)^a^						
compound	EC 25922	PA 9027	PΔ6	BAA 2108	BAA 2109	BAA 2110	PA ATP synthesis IC_50_ (μg/mL)
**1**(18)	>128	>256	>256	—	—	—	10.0
**4**	256	>256	64–128	128–256	256	>256	11.1
**5**	64	>128	16	64	>128	>128	0.7
gentamicin	—	2–4	>4	16	2	4–8	—

a *n* = 3, MIC = minimum inhibitory concentration of >85% reduction in pathogen growth with compound compared to pathogen alone (DMSO only) at OD 590 nm (no visible growth).

With confirmation that compounds **4** and **5** are capable of inhibiting ATP synthesis in PA vesicles, we then proceeded to evaluate the full antibiotic potential of these compounds. As discussed previously, although our initial SAR evaluation produced multiple molecules capable of inhibiting PA ATP synthase at low concentrations, none were able to act as antibiotics against whole-cell PA due to poor outer membrane penetration and/or rapid efflux from the cell.^18^ The dimethylamine group on compounds **4** and **5** is readily protonated and greatly increases the overall solubility of both molecules, with compound **4** being fully soluble and compound **5** being partially soluble in dimethyl sulfoxide (DMSO) at 256 μg/mL, but both being very soluble at 128 μg/mL. Therefore, we hypothesized that this increase in solubility and the ability for the amine to be protonated would allow compounds **4** and **5** to more readily cross the OM of PA compared to our prior set of molecules. We tested the antibacterial activity of compounds **4** and **5** against the EC 25922, PA 9027, and PΔ6 strains as well as 3 MDRPA clinical isolates (BAA 2108, BAA 2109, and BAA 2110) from cystic fibrosis patients that are broadly resistant to penicillin and cephalosporin antibiotics, tigecycline, and nitrofurantoin and are susceptible to quinolone and aminoglycoside antibiotics. The results are shown in Table 1 with compound **1** and gentamicin for reference. Compound **4** was able to weakly inhibit EC and the efflux knockout strain PΔ6; however, it was inactive against PA 9027. Against the MDR strains, compound **4** was able to inhibit BAA 2108 and BAA 2109, albeit at higher concentrations. Compound **5** was more potent against EC 25922, PΔ6, and BAA 2108, but it was inactive against PA 9027, BAA 2109, and BAA 2110. The inactivity of both compounds against the nonvirulent strain of PA (9027) compared to the efflux knockout strain (PΔ6) indicates that either these compounds are susceptible to efflux or are inhibited by biofilm formation in PA 9027. However, both compounds do appear to readily cross the OM. The activity of compounds **4** and **5** against MDRPA strains demonstrates that ATP synthase is a druggable target in PA that can result in inhibition of bacterial cell growth, though additional mechanisms of action cannot be definitively excluded.

### Expression of PA ATP Synthase in *E. coli*

**Figure 3:**
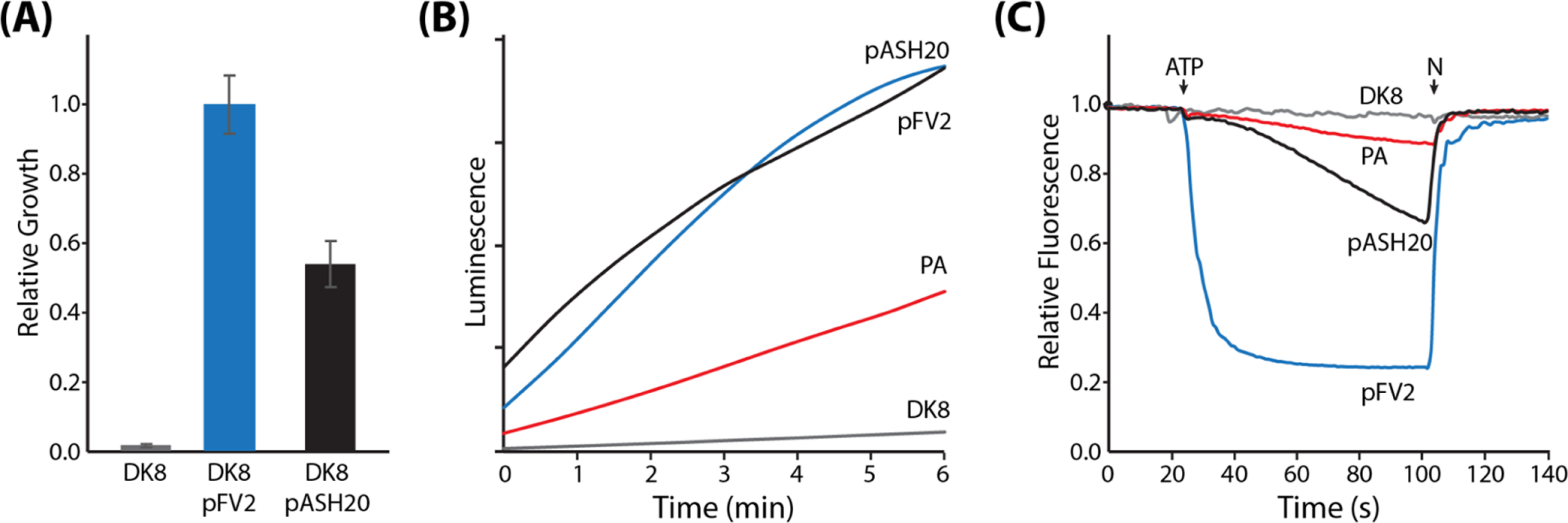
Functional characterization of PA ATP synthase expressed in *E. coli*. (A) *E. coli* DK8 cells transformed with pFV2,^20^ encoding the EC ATP synthase, or pASH20, encoding the PA ATP synthase, were grown in minimal medium containing succinate as the sole carbon source. Bars show mean maximum growth (measured as OD_550_) after 8 h normalized to that of DK8/pFV2. Error bars report standard deviation of *n* ≥ 3 replicates. (B) NADH-driven ATP synthesis activity of membrane vesicles (10 μg protein) from PA (red), *E. coli* DK8 (gray), DK8 pFV2 (blue), and DK8/pASH20 (black) was measured in real time using a luminescence assay as described in Methods section. Representative traces show raw luminescence in arbitrary units over time. (C) ATP-driven H^+^ pumping activity of inverted membrane vesicles (coloring as in panel B) was measured by quenching of ACMA fluorescence as described in Methodssection. Representative traces show fluorescence values normalized to *t* = 0, and the addition of ATP and nigericin (N) is indicated.

To validate ATP synthase as the target of these quinoline derivatives and facilitate further studies into the quinoline binding site in PA ATP synthase, we constructed the pASH20 plasmid to express PA ATP synthase in *E. coli* (Figure S1). This plasmid, derived from the commercially available pBR322 vector and containing the whole PA *atp* operon, was used to transform DK8, an EC K-12 strain lacking an endogenously encoded ATP synthase.^19^ The DK8/pASH20 strain was able to grow on minimal medium, where succinate is the sole carbon source (Figure 3A), which indicates the expression of a functional ATP synthase. Additionally, inverted membrane vesicles prepared from DK8/pASH20 showed ATP synthesis activity equivalent to that of DK8/pFV2, a strain with plasmid-encoded EC ATP synthase that is widely used as a baseline in biochemical studies (Figure 3B).^20−22^ ATP synthesis activity of PA vesicles was lower than either of the *E. coli* transformants, perhaps due to a higher abundance of ATP synthase when expressed from a plasmid. Additionally, since the expression of electron transport components in PA varies greatly depending on growth conditions,^23^ the *E. coli* membranes could be providing a stable amount of the electron transport complexes that drive the assay. Finally, we measured the ATP-driven H^+^-pumping activity of inverted membrane vesicles (Figure 3C) from the PA and EC strains. This assay tests the reverse activity of ATP synthase, which uses ATP hydrolysis to pump H^+^ into the lumen of the vesicle, where decreased interior pH quenches a fluorescent dye. PA vesicles have low activity relative to *E. coli* (DK8/pFV2), a phenotype observed previously in PA^15^ and shared by the closely related ATP synthase from *A. baumannii*.^17^ Consistent with the behavior of PA vesicles, vesicles prepared from DK8/pASH20 also showed reduced H^+^-pumping activity relative to DK8/pFV2.

### Inhibition of DK8/pASH20 ATP Synthesis Activity by Compounds **1**, **4**, and **5**

**Figure 4:**
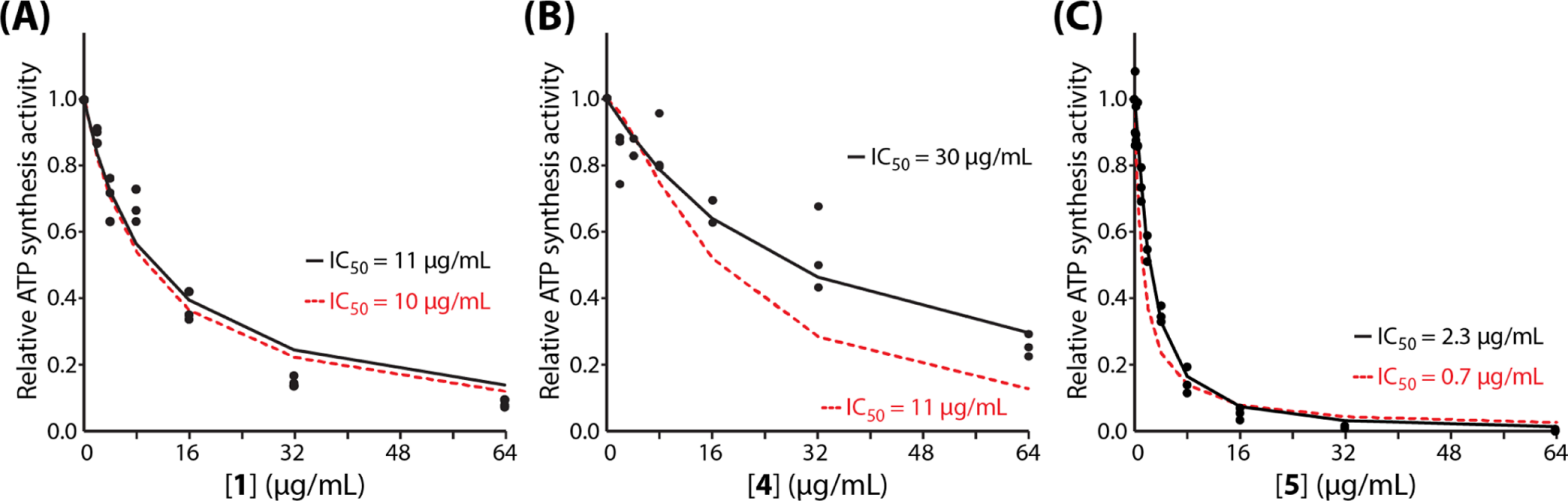
Inhibition of ATP synthesis activity in DK8/pASH20 membrane vesicles. NADH-driven ATP synthesis activity of inverted membrane vesicles from *E. coli* DK8/pASH20 was measured in the presence of 0–64 μg/mL compound **1** (A), **4** (B) or **5** (C) using an end point luminescence assay as described in Methods section. Luminescence values in each replicate (black dots) were normalized to 0 μg/mL compound, and mean values were used to fit a dose–response curve (black line). The *dotted red line* in each panel is the dose–response fit for PA vesicles from Figure 2 for reference.

Using inverted membrane vesicles prepared from the DK8/pASH20 strain, we tested the inhibition of NADH-driven ATP synthesis activity by compounds **1**, **4**, and **5** (Figure 4). Compound **1** inhibited activity with IC_50_ values similar to those determined for PA vesicles (10.5 μg/mL). The IC_50_ value for compound **4** (30.3 μg/mL) was greater than that determined for PA vesicles, and compound 5 had a slightly increased IC_50_ value (2.3 μg/mL). The larger deviation for compound **4** is likely due to additional off-target inhibition of the PA electron transport chain by this compound (Table 2 and Figure S2). Compound **1** did not inhibit the electron transport chain strongly in either PA or EC, and the off-target inhibition of the PA electron transport chain by compound **5** was much weaker than the inhibition of ATP synthase (Table 2 and Figure S2). The similarity of ATP synthesis inhibition by these compounds in vesicles prepared from PA and DK8/pASH20 is evidence that PA ATP synthase is the target of these inhibitors since a similar IC_50_ was observed when PA ATP synthase was isolated from the other components of the PA membrane. Interestingly, the C2 dimethylamine substituent of compounds **4** and **5** seem to enable inhibition of some component of the PA electron transport chain, whereas the EC electron transport chain was not strongly affected. Since the bacterial growth conditions used in this study did not control for the complex expression patterns of PA respiratory complexes,^24^ we cannot determine what NADH-driven H^+^ pumping activity was affected.

**Table 2:** Inhibition of the PA and EC electron transport chains

	IC_50_ (μg/mL)	
compound	PA	EC (DK8/pASH20)
**1**	>64^18^	>64
**4**	29	61
**5**	27	>64

### Mutations in the H^+^ Binding Site of PA ATP Synthase Altered Inhibition by **4** and **5**

**Figure 5:**
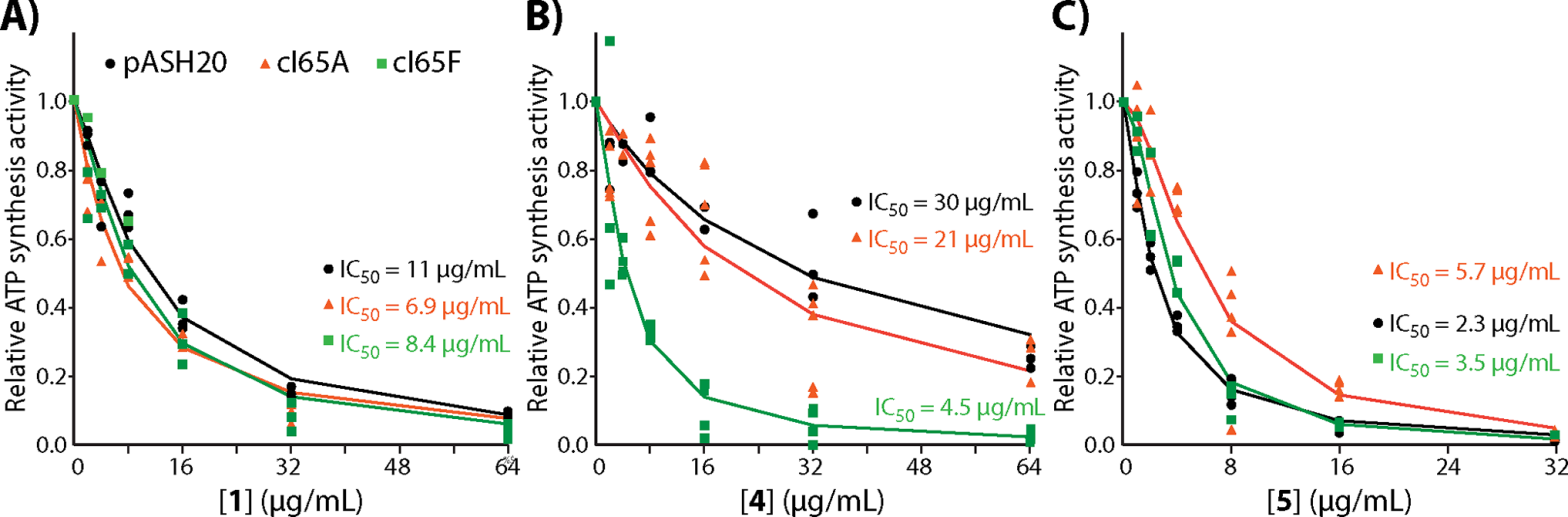
Inhibition of ATP synthesis activity in mutant DK8/pASH20 membrane vesicles. NADH-driven ATP synthesis activity of inverted membrane vesicles from WT (black), *c*I65A (*orange*), or *c*I65F (*green*) was measured in the presence of 0–64 μg/mL compound **1** (A) or **4** (B), or 0–32 μg/mL compound **5** (C) using a luminescence assay as described in Methods section. Luminescence values in each replicate (dots) were normalized to 0 μg/mL compound, and mean values were used to fit a dose–response curve (lines).

BDQ is known to bind the *c*-ring of MT ATP synthase at the essential acidic residue, which functions as the H^+^ binding site. To confirm that our quinoline derivatives also target the H^+^ binding site in PA ATP synthase, we mutated Ile65 in subunit *c* (Figure 1B) to Ala or Phe and observed the effect of these mutations on ATP synthesis inhibition. Mutations were introduced to the atpE gene in pASH20 by using a synthetic gene fragment. The *c*Ile65Ala and *c*Ile65Phe mutations still support ATP synthesis activity *in vivo* and *in vitro* (Figure S3). We tested the inhibition of the NADH-driven ATP synthesis activity of inverted vesicles from mutant DK8/pASH20 by compounds **1**, **4**, and **5**. The *c*Ile65 mutations did not significantly alter the inhibition by compound **1** (Figure 5A). The absence of an effect does not exclude the possibility that **1** binds near this site in a mode that does not contact Ile65. In contrast, compound **4** showed increased potency against the *c*Ile65Phe mutant (Figure 5B) with a greater than 6-fold reduction in IC_50_, and the *c*Ile65Ala mutation decreased the potency of compound **5** (Figure 5C). The marked increase in affinity of **4** for the Phe mutant likely resulted from the creation of a new protein–ligand contact, perhaps enhancing pi stacking with the quinoline. Oppositely, the Ala mutation likely removes a contact from compound **5**, perhaps reducing van der Waals interactions with the C1 benzyl sulfide. The surface location of Ile65 (Figure 1B) as well as the lack of significant functional effects make it unlikely that these mutations are propagating a structural change that would alter binding at a distant site. Therefore, the alterations of apparent binding affinity suggest that these quinoline compounds bind to the *c*-ring in the region of the H^+^ binding site.

Additionally, we observed that the mutations increase the Hill coefficients of the dose–response curves (Table S2), which could indicate that the quinoline compounds occupy more than one binding site. Since there are 10 *c* subunits in the *c*-ring (based on the stoichiometry in *A. baumannii*(17)), there are potentially 10 binding sites for the quinolines. Structures of MT ATP synthase bound to BDQ^12^ indicated that BDQ can bind to multiple H^+^ binding sites around the *c*-ring, with the “leading” site at the interface of subunit *a* and the *c*-ring having the highest affinity. The increase in Hill slope could result from (1) reduced binding affinity at a higher affinity site such that multiple sites have more comparable occupancies (fits the effect of the *c*I65A mutation on inhibition by compound **5**) or (2) increased binding affinity at all sites around the *c*-ring so that occupancy increases (fits the effect of the *c*I65F mutation on inhibition by compound **4**). Further studies will be required to definitively determine the quinoline binding site and stoichiometry of the PA *c*-ring.

## Conclusions

In previous work,^18^ we conducted an initial SAR study of quinolines derivatized at the C1 and C2 positions and their inhibition of PA ATP synthesis activity, and we found that bulky constituents at C1 and C2 and hydrogen bonding capability at C2 related to stronger inhibition. Based on those results, we generated additional quinoline derivatives that maintained (methyl sulfide) or increased (benzyl sulfide) hydrophobic bulk at C1 and introduced bulky constituents at C2. Characterization of these compounds showed that **4** and **5** were the most potent inhibitors of PA ATP synthesis activity *in vitro*, and compound **5** had antibiotic activity against a drug-resistant clinical isolate of PA, though a lower MIC against PA PΔ6 indicates that **5** is effluxed from the cell. These results suggest that additional hydrophobic bulk at C1 and a dimethylamine at C2 are promising directions for subsequent derivations. In addition to these novel compounds, we have generated a whole-operon expression plasmid for PA ATP synthase in *E. coli*, which will facilitate further study of the structure, function, and inhibition of this enzyme. Using this expression system, we showed via mutagenesis that compounds **4** and **5** likely bind to the *c*-ring of PA ATP synthase near the H^+^ binding site. Therefore, their mechanism of action is likely similar to that of BDQ against MT ATP synthase. Overall, this study reports chemical and genetic tools to aid the discovery of new antibiotics against drug-resistant PA by targeting ATP synthase.

## Methods

### Synthesis and Spectroscopic Data

#### General

Reagents and solvents were purchased as reagent grade and used without further purification. All reactions were performed in flame-dried glassware under an Ar or N_2_ atmosphere. Evaporation and concentration *in vacuo* were performed at 40 °C. TLC was conducted using precoated SiO_2_ 60 F254 glass plates from EMD with visualization by UV light (254 or 366 nm). NMR (^1^H or ^13^C) spectra were recorded on a Varian INOVA-400 MHz spectrometer at 298 K. Residual solvent peaks were used as an internal reference. Coupling constants (*J*) (H,H) are given in Hz. Coupling patterns are designated as singlet (s), doublet (d), and triplet (t). IR spectra were recorded on a Shimadzu IR Spirit FT-IR spectrophotometer and measured without solvent. Low-resolution mass spectral data were acquired on a Shimadzu single quadrupole LCMS-2020. High-resolution mass spectral samples were analyzed with a Q Exactive HF-X (ThermoFisher, Bremen, Germany) mass spectrometer. Samples were introduced via a heated electrospray source (HESI) at a flow rate of 10 μL/min. HESI source conditions were set as nebulizer temperature 400 °C, sheath gas (nitrogen) 20 arb, auxiliary gas (nitrogen) 0 arb, sweep gas (nitrogen) 0 arb, capillary temperature 320 °C, and RF voltage 45 V. The mass range was set to 100–1000 *m*/*z*. All measurements were recorded at a resolution setting of 120,000. Solutions were analyzed at 0.1 mg/mL or less based on responsiveness to the ESI mechanism. Xcalibur (ThermoFisher, Breman, Germany) was used to analyze the data. Molecular formula assignments were determined with the Molecular Formula Calculator (v 1.3.0). All observed species were singly charged, as verified by unit *m*/*z* separation between mass spectral peaks corresponding to the ^12^C and ^13^C^12^C_c-1_ isotope for each elemental composition.

#### Safety Statement

No chemical safety hazards were encountered during the synthetic experiments of this research work.

##### 2-(Benzylthio)quinoline-3-carbaldehyde **3**

2-Chloroquinoline-3-carbaldehyde (2.0 g, 10.4 mmol) and Na_2_S (1.96 g, 25.0 mmol) were dissolved in *N,N*-dimethylformamide (0.2 M) and allowed to stir at 23 °C for 15 h. Then, K_2_CO_3_ (2.16 g, 15.6 mmol) and benzyl bromide (1.86 mL, 15.6 mmol) were added, and the solution was warmed to 100 °C for 2 h. After 2 h, the reaction was cooled to 23 °C and diluted with DI H_2_O. The solution was then extracted with ethyl acetate (3×). The organic layers were then combined and concentrated under reduced pressure. Flash chromatography of the crude extracts (SiO_2_, 5 × 15 cm, 5–10% ethyl acetate/hexanes gradient elution) provided the desired product **3** as a yellow solid (1.42 g, 49%). ^1^H NMR (CDCl_3_, 400 MHz): δ 10.10 (s, 1H), 8.20 (s, 1H), 7.93 (d, *J* = 8.8 Hz, 1H), 7.72 (t, *J* = 7.2 Hz, 1H), 7.68 (d, *J* = 8.4 Hz, 1H), 7.50 (d, *J* = 6.8 Hz, 2H), 7.41 (t, *J* = 7.6 Hz, 1H), 7.28 (t, *J* = 7.6 Hz, 2H), 7.20 (t, *J* = 7.2 Hz, 1H), 4.56 (s, 2H). ^13^C NMR (CDCl_3_, 100 MHz): δ 189.92, 158.46, 149.24, 142.86, 137.95, 133.08, 129.54, 129.23, 128.46, 127.93, 127.13, 126.87, 126.23, 124.51, 34.03. IR (film): *v*_max_ 2923, 1691, 1614, 1584, 1584, 1548, 1494, 1452, 1386, 1328, 1172, 1135, 1055, 764, 744, 695 cm^–1^. HRMS (ESI) *m*/*z* [M + H]^+^: calcd for C_17_H_14_NOS 280.0791; found: 280.07834.

##### 1-(4-((((2-(Benzylthio)quinolin-3-yl)methyl)amino)methyl)phenyl)-*N*,*N*-dimethylmethanamine **5**

2-(Benzylthio)quiline-3-carbaldehyde **3** (100 mg, 0.35 mmol) and 1-(4-(aminomethyl)phenyl)-*N,N*-dimethylmethanamine hydrochloride (86 mg, 0.43 mmol) were dissolved in anhydrous methanol (4 mL, 0.09 M) under inert conditions. *N,N*-Diisopropylethylamine (0.19 mL, 1.1 mmol) was then added dropwise, and the reaction mixture was allowed to stir at 23 °C for 24 h. NaBH_4_ (27 mg, 0.71 mmol) was then added. After 1 h, the reaction mixture was diluted with DI H_2_O and extracted with dichloromethane (2x). The organic layers were then combined, dried in Na_2_SO_4_, and concentrated under reduced pressure. Flash chromatography of the crude extracts (SiO_2_, 3 × 10 cm, 0–10% CH_3_OH/CH_2_Cl_2_ gradient elution) provided the desired product **5** as a pale yellow semisolid (0.123 g, 72%). ^1^H NMR (CDCl_3_, 400 MHz): δ 7.98 (d, *J* = 8.4 Hz, 1H), 7.92 (s, 1H), 7.70 (d, *J* = 7.2 Hz, 1H), 7.62 (t, *J* = 8.4 Hz, 1H), 7.48 (d, *J* = 7.6 Hz, 2H), 7.41 (t, *J* = 8.0 Hz, 1H), 7.33–7.21 (m, 7H), 4.65 (s, 2H), 3.88 (s, 2H), 3.80 (s, 2H), 3.52 (s, 2H), 2.30 (s, 6H). ^1^H NMR (acetone-d6, 400 MHz): δ 8.21 (s, 1H), 7.99 (d, *J* = 7.6 Hz, 1H), 7.87 (d, *J* = 8.8 Hz, 1H), 7.68 (t, *J* = 8.4 Hz, 1H), 7.53 (d, *J* = 8.0 Hz, 2H), 7.48 (t, *J* = 8.0 Hz, 1H), 7.40 (m, 4H), 7.29 (t, *J* = 8.4 Hz, 2H), 7.21 (t, *J* = 7.2 Hz, 1H), 4.64 (s, 2H), 3.88 (s, 2H), 3.87 (s, 2H), 3.67 (s, 2H), 2.34 (s, 6H). ^13^C NMR (CDCl_3_, 100 MHz): δ 158.21, 147.04, 139.44, 138.37, 135.54, 133.93, 131.05, 129.65, 129.34, 129.21, 128.49, 128.35, 127.66, 127.48, 127.10, 126.05, 125.38, 63.43, 52.97, 49.48, 44.71, 34.05. IR (film): ν_max_ 2920, 2851, 2815, 2769, 1647, 1596, 1494, 1453, 1396, 1327, 1135, 1043, 751, 696 cm^–1^. HRMS (ESI) *m*/*z* [M + H]^+^: calcd for C_27_H_30_N_3_S 428.2155; found: 428.21480.

##### N,N-Dimethyl-1-(4-((((2-(methylthio)quinolin-3-yl)methyl)amino)methyl)phenyl)methanamine **4**

2-(Methylthio)quinoline-3-carbaldehyde^18^**2** (151 mg, 0.74 mmol) and 1-(4-(aminomethyl)phenyl)-*N,N*-dimethylmethanamine hydrochloride (179 mg, 0.89 mmol) were dissolved in anhydrous methanol (9 mL, 0.09 M) under inert conditions. *N,N*-Diisopropylethylamine (0.39 mL, 2.2 mmol) was then added dropwise, and the reaction mixture was allowed to stir at 23 °C for 16 h. NaBH_4_ (60 mg, 1.5 mmol) was then added. After 1 h, the reaction mixture was diluted with DI H2O and extracted with ethyl acetate. The organic layer was washed with saturated aqueous NaCl, dried over Na_2_SO_4_, and concentrated under reduced pressure. Flash chromatography of the crude extracts (SiO_2_, 1 × 10 cm, 0–5% CH_3_OH/CH_2_Cl_2_ gradient elution) provided the desired product **4** as a yellow foam (123 mg, 47%). ^1^H NMR (CDCl_3_, 400 MHz): δ 7.96–7.92 (m, 2H), 7.73 (d, *J* = 8 Hz,1H), 7.62 (t, *J* = 7.6 Hz, 1H), 7.42 (t, *J* = 7.6 Hz, 1H), 7.38–7.32 (m, 4H), 3.95 (s, 2H), 3.85 (s, 2H), 3.57 (s, 2H), 2.72 (s, 3H), 2.34 (s, 6H). ^13^C NMR (CDCl_3_, 100 MHz): δ 159.25, 147.34, 139.62, 135.44, 133.81, 131.17, 129.82, 129.24, 128.50, 127.75, 127.57, 125.90, 125.36, 63.51, 53.07, 49.69, 44.75, 13.06. IR (film): ν_max_ 3303, 3046, 3023, 2970, 2921, 2852, 2809, 2760, 1613, 1595, 1556, 1511, 1488, 1453, 1394, 1361, 1326, 1310, 1254, 1203, 1169, 1134, 1096, 1040, 1030, 1017, 953, 904, 855, 800, 777, 747, 680, 618, 565, 520, 501, 480 cm^–1^. HRMS (ESI) *m*/*z* [M + H]^+^: calcd for C_21_H_26_N_3_S 352.1842; found: 352.18352.

### Biological Evaluation of Lead Compounds

#### General Sterilization Procedure

The following are general steps, unless otherwise noted. All steps were completed using aseptic techniques. All media and glassware were sterilized via an autoclave at 121 °C for 60 min. All agitation occurred at 160 rpm in a temperature-controlled console shaker (Excella E25) at 37 °C. Full-strength tryptic soy broth (TSB) was made by dissolving 30 g of BD Bacto TSB powder in 1 L of deionized water. Purchased and acquired bacterial strains used were *E. coli* (ATCC 25922), *P. aeruginosa* (ATCC 9027), drug-resistant *P. aeruginosa* from ATCC Panel MP-23 (BAA 2108, BAA 2109, BAA 2110), and *P. aeruginosa* efflux knockout GKCW120 (PΔ6)^6^ provided by Zgurskaya and co-workers at the University of Oklahoma.

#### Safety Statement

**Caution!** Experiments utilizing *P. aeruginosa* strains were handled following BSL 2 protocols. **Caution!** CCCP and nigericin are ionophores that are toxic if swallowed.

#### Antimicrobial Susceptibility Assay Procedure

Susceptibility testing was performed in biological triplicate using the broth microdilution method as outlined by the Clinical and Laboratory Standards Institute. Briefly, minimum inhibitory concentration (MIC) determinations were carried out in 96-well microtiter plates with 2-fold serial dilutions of the compounds from 0 to 256 μg/mL (final assay concentrations) in DMSO. Briefly, to each well, 1 μL of compound in DMSO, 89 μL of TSB, and 10 μL of bacterial inoculum, grown from a single colony in 10 mL of TSB for 4–6 h, were added. After incubation for 12–15 h at 37 °C, absorbance at 590 nm was read on a Biotek Synergy HTX Multimode plate reader. Data were processed by background subtracting the media absorbance and then normalizing the data to full bacterial growth with the vehicle only. MIC is defined as the lowest concentration of antibiotic or antibiotic/adjuvant combination that achieves ≥85% growth inhibition, which corresponds to no visible growth.

#### Construction of pASH20 Expression Plasmid and Derivatives

*P. aeruginosa* (ATCC 9027) was streaked onto a 10% TSB agar plate and incubated at room temperature for 72 h. A single colony was resuspended in 100 μL of H_2_O, and 5 μL was used as the DNA template for PCR with Q5 polymerase (NEB) and 0.25 mM each of forward and reverse primers. Primers were complementary to regions flanking the *atp* operon and included sequences for *Hin*dIII and NdeI restriction sites (see the SI for primer sequences). The resulting 6.9 kbp DNA fragment was digested with *Hin*dIII and NdeI and ligated into digested vector pBR322 (New England Biolabs). The resultant pASH20(-i) plasmid contains a partial *atpI* gene. To complete the *atpI* gene, the *HindII-XhoI* fragment of pASH20(-i) was replaced with a synthetic fragment with a complete *atpI* (Twist Bioscience) to yield pASH20. The sequence of the entire operon was verified by Sanger DNA sequencing through the ligation sites. Subunit *c* mutations were introduced by exchanging the *XhoI-BamHI* fragment containing *atpE* with a synthetic fragment containing mutated *atpE* (Twist Bioscience).

#### Preparation of Inverted Membrane Vesicles

Vesicles were prepared from *P. aeruginosa* and transformed *E. coli* cells as previously described.^22^ Briefly, cells were grown in LB liquid medium at 37 °C with shaking and harvested by centrifugation after 7 h of growth. Cultures of transformant *E. coli* included 100 μg/mL ampicillin. Cells were disrupted in TMG buffer (50 mM Tris-HCl, MgCl_2_, 10% (v/v) glycerol, pH 7.5) using an Avestin B15 homogenizer at 19,000 psi. After unbroken cells and debris were cleared by centrifugation at 9000*g*, inverted vesicles were collected by centrifugation at 193,000*g*, resuspended in TMG buffer, and stored at −80 °C. Protein concentrations were determined using a modified Lowry assay.^25^

#### Determination of ATP Synthesis Activity

Continuous ATP synthesis activity of inverted membrane vesicles was measured using a luciferin/luciferase luminescence assay as previously described.^22^ Inhibition of ATP synthesis activity by test compounds was determined using an end point assay essentially as previously described.^18,26^ In this assay, the synthesis reaction was initiated by 2.5 mM NADH, proceeded for 10 min, and was stopped with 1% trichloroacetic acid, 2 mM CCCP, and 10 mM EDTA. Samples from each reaction were diluted 500-fold prior to the addition of luciferase since quinoline compounds are known to inhibit luciferase.^27^ This dilution was sufficient to minimize inhibition of luciferase (Figure S4). Each replicate set included a positive control containing DMSO with no compound and a negative control containing carbonyl cyanide *m*-chlorophenyl hydrazone. Luminescence values were corrected for the background by subtracting the negative control and then normalized to the positive control within the same replicate. Dose–response curves were fit using eq 1, where activity is the relative ATP synthesis activity, [I] is the concentration of inhibitor in μg/mL, and *n* is the Hill coefficient.
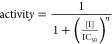
1

#### Growth of Transformant *E. coli* on Succinate Minimal Medium

As previously described,^22^ transformant *E. coli* DK8 strains were streaked on an LB agar plate containing 100 μg/mL ampicillin, and a single colony was used to inoculate 5 mL M63-TIV medium (61.8 mM KH_2_PO_4_, 38.2 mM K_2_HPO_4_, 15 mM (NH_4_)_2_SO_4_, 1 mM MgSO_4_, 1 μg/mL thiamine, 0.2 mM isoleucine, 0.2 mM valine) containing 0.1% (w/v) glucose and 5% (v/v) LB and incubated overnight at 37 °C with shaking. Wells in a clear 96-well plate with M63-TIV medium (250 μL) containing 0.6% (w/v) sodium succinate and ampicillin were inoculated with 5 μL of dense culture, and growth over 10 h at 37 °C was measured by OD_550_. Untransformed DK8 control wells did not contain ampicillin.

#### Determination of ATP-Driven H^+^ Pumping Activity

As described previously,^22^ inverted membrane vesicles (1.6 mg protein) were added to HMK buffer (10 mM HEPES-KOH, 1 mM MgCl_2_, 300 mM KCl, pH 7.5) containing 0.3 μg/mL 9-amino-6-chloro-2-methoxyacridine (ACMA), and fluorescence (λ_ex_ = 415 nm, λ_em_ = 485 nm) was monitored in a Shimadzu RF-6000 spectrofluorometer with magnetic stirring. H^+^ pumping was initiated at *t* = 20 s by the addition of ATP to 0.25 mM, and the H^+^ gradient was equilibrated at *t* = 100 s by the addition of nigericin to 0.5 μg/mL.

## Supporting Information Available

SI includes synthesis and characterization of compounds **S1**-**S6** (Schemes S1 and S2), initial compound screening results (Table S1), ^1^H and ^13^C NMR spectra for **4** and **5**, details of primer design for cloning of the PA atp operon and a map of the pASH20 plasmid (Figure S1), fit parameters for inhibition curves (Table S2), and control experiments for inhibition of the electron transport chain (Figure S2), function of subunit c mutants (Figure S3), and inhibition of luciferase (Figure S4) (PDF)

## Abbreviations

ATP: adenosine triphosphate
ETC: electron transport chain
PA: *Pseudomonas aeruginosa*
MT: *Mycobacterium tuberculosis*
EC: *Escherichia coli*
MDR: multidrug resistant
MIC: minimum inhibitory concentration
IC_50_: inhibitory concentration of 50%
SAR: structure–activity relationship
DMSO: dimethyl sulfoxide
DCM: dichloromethane
THF: tetrahydrofuran
TSB: tryptic soy broth
