# Supplementary material for: Quinoline Compounds Targeting the c-Ring of ATP Synthase Inhibit Drug-Resistant Pseudomonas aeruginosa
Source: ACS Infect Dis. 2023 Nov 3;9(12):2448–56. doi: 10.1021/acsinfecdis.3c00317 (PMC10714390; doi:10.1021/acsinfecdis.3c00317)
Supplement: Supplementary file 1 — id3c00317_si_001.pdf [file id3c00317_si_001.pdf]

SUPPORTING INFORMATION

Quinoline compounds targeting the *c*-ring of ATP synthase inhibit drug-resistant *Pseudomonas aeruginosa*

Authors:

Vesper M. Fraunfelter,[1] Bryce A. Pugh,[1] Alexander P. L. Williams,[1] Katie T. Ward,[1] Dietrich O. Jackson,[1] Molly Austin,[1] John F. Ciprich,[1] Lorelei Dippy,[1] Jason Dunford,[1] G. Nathaniel Edwards,[1] Evan Glass,[1] Kyle M. Handy,[1] Casey N. Kellogg,[1] Kaitlyn Llewellyn,[1] K. Quinn Nyberg,[1] Sam J. Shepard,[1] Casey Thomas,[1] Amanda L. Wolfe,[1]* P. Ryan Steed[1]*

[1]Department of Chemistry and Biochemistry, University of North Carolina Asheville, One University Heights, Asheville, North Carolina, 28804, United States

*Corresponding authors: P. Ryan Steed, psteed@unca.edu; Amanda L. Wolfe, awolfe@unca.edu

Table of Contents

Synthesis and Spectroscopic Data of Inactive Compounds S2

Scheme S1. Synthesis of compound S1-S4 via reductive amination. S2

Scheme S2. Synthesis of compounds S5-S6. S4

Initial antibacterial activity screen of compounds 4, 5, and S1-S6 S6

Table S1. Antibacterial activity screening results for compounds 4, 5, and S1-S6 at 128 ug/mL S6

NMR spectra for compounds 4 and 5 S7

Primers for PA ATP synthase amplification S9

Figure S1. Plasmid map of pASH20 S9

Figure S2. Inhibition of PA and EC electron transport chain (ETC) S10

Figure S3. Growth and Activity of DK8/pASH20 Ile65 Mutants S10

Table S2. All IC_{50} values and Hill coefficients S11

Figure S4. Inhibition of luciferase by quinoline compounds S11

Supplemental Methods S12

Supplemental References S12

Synthesis and Spectroscopic Data of Inactive Compounds

General. Reagents and solvents were purchased reagent-grade and used without further purification. All reactions were performed in flame-dried glassware under an Ar or N_2 atmosphere. Evaporation and concentration *in vacuo* was performed at 40 °C. TLC was conducted using precoated SiO_2 60 F254 glass plates from EMD with visualization by UV light (254 or 366 nm). NMR (^{1}H or ^{13}C) were recorded on an Varian INOVA-400 MHz spectrometer at 298 K. Residual solvent peaks were used as an internal reference. Coupling constants (*J*) (H,H) are given in Hz. Coupling patterns are designated as singlet (s), doublet (d), triplet (t), broad singlet (br), or doublet of doublets (dd). IR spectra were recorded on a Shimadzu IRSpirit FT-IR spectrophotometer and measured neat. Low-resolution mass spectral data were acquired on a Shimadzu single quadrupole LCMS-2020. High-resolution mass spectral Samples were analyzed with a Q Exactive HF-X (ThermoFisher, Bremen, Germany) mass spectrometer. Samples were introduced via a heated electrospray source (HESI) at a flow rate of 10 µL/min. HESI source conditions were set as: nebulizer temperature 400 deg C, sheath gas (nitrogen) 20 arb, auxiliary gas (nitrogen) 0 arb, sweep gas (nitrogen) 0 arb, capillary temperature 320 degrees C, RF voltage 45 V. The mass range was set to 100-1000 m/z. All measurements were recorded at a resolution setting of 120,000. Solutions were analyzed at 0.1 mg/mL or less based on responsiveness to the ESI mechanism. Xcalibur (ThermoFisher, Breman, Germany) was used to analyze the data. Molecular formula assignments were determined with Molecular Formula Calculator (v 1.3.0). All observed species were singly charged, as verified by unit *m/z* separation between mass spectral peaks corresponding to the ^{12}C and $^{13}C^{12}C_{c-1}$ isotope for each elemental composition.

Synthesis of Analogs S1-S4

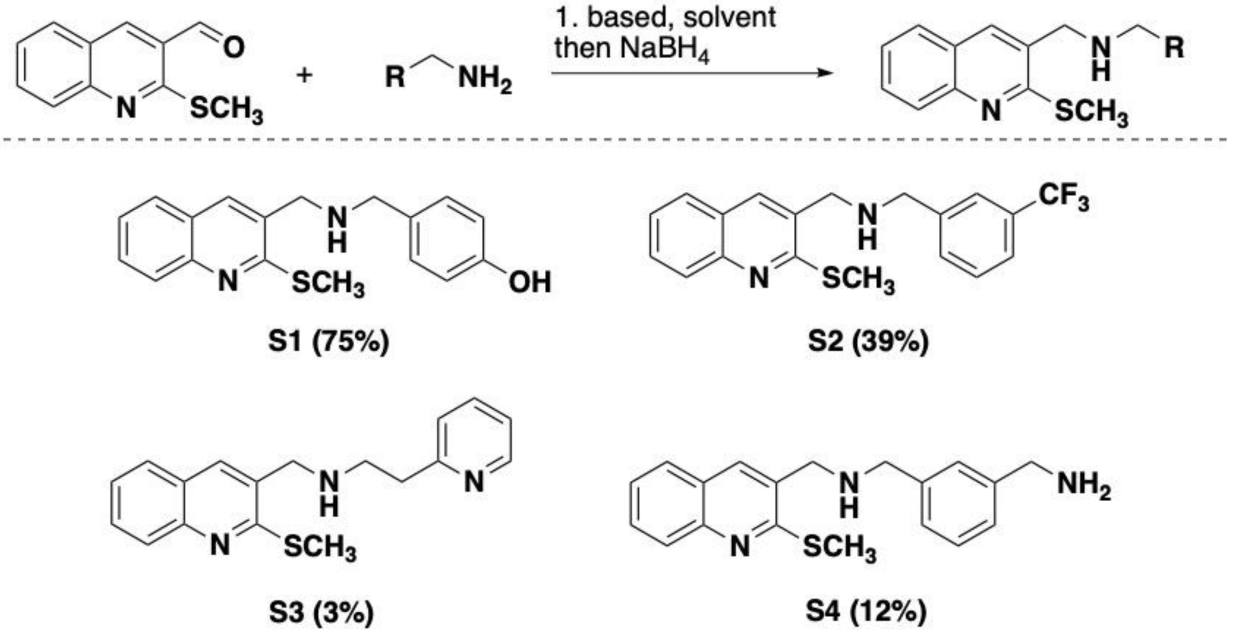

Scheme S1. Synthesis of compound S1-S4 via reductive amination.

***4-((((2-(methylthio)quinolin-3-yl)methyl)amino)methyl)phenol* (S1).**

2-(methylthio)quinoline-3-carbaldehyde (0.150 g, 0.80 mmol) and 4-(aminomethyl)phenol (0.108 g, 0.88 mmol) were dissolved in *N,N*- Diisopropylethylamine (0.38 mL) and methanol (8.1 mL) and allowed to stir at 23 °C overnight under nitrogen atmosphere. Then, sodium borohydride (0.0550 g, 1.460 mmol) was added, and the reaction was allowed to stir at 23 °C and monitored for completion using thin-layer chromatography. After completion, the reaction was diluted with DI H_2O and extracted with dichloromethane. The organic layer was washed with saturated aqueous NaCl, dried over Na_2SO_4, and concentrated under reduced pressure. Flash chromatography of the crude extracts (SiO_2, 2 x 10 cm, 25% EtOAc/hexane elution) yielded molecule **S1** (0.169 g, 75%) as brown crystals. ^{1}H NMR ($CDCl_3$, 400 MHz): δ 7.95-6.78 (m, 1H), 7.76 - 7.57 (m, 3H), 7.36-7.32 (m, 1H), 7.27 - 7.03 (m, 2H), 6.78 (br, 2H), 4.00 - 3.51 (m, 4H), 2.70 - 2.61 (m, 3H). ^{13}C NMR ($CDCl_3$, 100 MHz): δ 159.98, 147.59, 131.63, 130.55, 130.32, 129.80, 127.75, 127.60, 125.83, 125.52, 115.89, 115.54, 115.35, 60.72, 29.88, 14.35, 13.62, 13.36, 13.14. IR (film) vmax 2925, 2843, 1703, 1613, 1596, 1556, 1513, 1490, 1450, 1395, 1330, 1313, 1248, 1208, 1169, 1135, 1095, 1049, 960, 912, 853, 823, 780, 750, 730, 600, 511, 480 cm^{-1}. HRMS (ESI) m/z [M+H]+: calcd for $C_{18}H_{19}N_2OS$ 311.1213; found: 311.12038.

***1-(2-(methylthio)quinolin-3-yl)-N-(3-(trifluoromethyl)benzyl)methanamine* (S2).**

2-(methylthio)quinoline-3-carbaldehyde (0.150 g, 0.74 mmol) and 3-(trifluoromethyl)benzylamine (0.11 mL, 0.74 mmol,) were dissolved in anhydrous methanol (8.1 mL, 0.09M). Then *N,N*-Diisopropylethylamine (0.38 mL, 2.19 mmol) was added and the reaction was warmed to 78 °C and allowed to react for 20 h. After 20 h, the reaction was then removed from heat, and allowed to cool to room temperature. $NaBH_4$ (60 mg, 1.476 mmol) was then added to the reaction and left to stir for 1 hour. Flash chromatography of the crude extracts (SiO_2, 3 × 15 cm, 16−50% EtOAc/hexane gradient elution) provided the desired product **S2** as a yellow oil (104 mg, 39%). ^{1}H NMR ($CDCl_3$, 400 MHz): δ 7.98 (d, *J* = 8.0 Hz, 1H), 7.88 (s, 1H) 7.74-7.42 (m, 7H), 4.74 (br, 1NH), 3.94 (s, 2H), 3.90 (s, 2H), 2.74 (s, 3H). ^{13}C NMR ($CDCl_3$, 100 MHz): δ 159.23, 147.42, 141.05,133.99, 132.41, 131.70, 131.61, 130.99, 130.89, 130.67, 130.35, 129.37, 129.01, 127.79, 127.75, 127.57, 125.92, 125.87, 125.73, 125.44, 125.38, 125.08, 124.97, 124.93, 124.15, 124.11, 124.08, 124.04, 123.02, 52,79, 49.77, 13,05, 12.809. IR (film) vmax: 3311, 3060, 2926, 2833, 1614, 1597, 1326, 1161, 1070, 1045, 749 cm^{-1}. HRMS (ESI) *m/z* [M+H]+: calcd for $C_{19}H_{18}N_2SF_3$ 363.1137; found: 363.11280.

***N-((2-(methylthio)quinolin-3-yl)methyl)-2-(pyridin-2-yl)ethan-1-amine* (S3).**

2-(methylthio)quinoline-3-carbaldehyde (0.2 g, 0.984 mmol) and 2-(pyridin-2-yl)ethan-1-amine (0.144 g, 1.181 mmol) were dissolved in anhydrous methanol (10.94 ml, 0.09 M) under inert conditions using sonication. *N,N*- Diisopropylethylamine (0.514 ml, 2.952 mmol) was added to the reaction mixture and left to stir at 23°C for 18-22 hours. $NaBH_4$ (0.0745 g, 1.968 mmol) was then added and left to stir for an hour. The reaction was then quenched with deionized (DI) H_2O and extracted with ethyl acetate. The organic layer was washed with saturated aqueous NaCl, dried over Na_2SO_4, and concentrated under reduced pressure. Flash chromatography of the crude extracts (SiO_2, 1 × 10 cm, 16−100% EtOAc/hexane gradient elution) provided the desired product **S3** as a yellow oil (8.2 mg, 3%). ^{1}HNMR ($CDCl_3$, 400 MHz): δ 8.22 (d, *J* = 5.2 Hz, 1H), 8.00-7.91 (m, 2H), 7.73-7.64 (m, 2H), 7.52-7.46 (m, 2H), 7.07-7.02 (m, 2H), 6.35 (s, 1H), 4.40 (d, J = 19.6 Hz, 1H), 3.83 (dd, J_1= 14.60 Hz, J_2= 9.20 Hz , 1H), 3.68-3.61 (m, 1H), 3.29 (dd, J_1=

26.0 Hz, J_2= 10.8 Hz, 1H), 2.98-2.96 (m, 1H), 2.07 (s, 3H). ^{13}CNMR ($CDCl_3$, 100 MHz): δ 159.25, 148.70, 137.77, 136.66, 130.20, 127.81, 125.73, 123.42, 121.60, 57.73, 52.43, 32.09, 13.15. IR (film) vmax: 3902, 3853, 3744, 3735, 3677, 3649, 2925, 2369, 1734, 1718, 1701, 1684, 1669, 1654, 1616, 1598, 1559, 1507, 1490, 1457, 1437, 1396, 1332, 1313, 1165, 1136, 1053 cm^{-1}. HRMS (ESI) *m/z* [M+H]+: calcd for $C_{18}H_{20}N_3S$ 310.1372; found: 310.13664.

***N-(3-(aminomethyl)benzyl)-1-(2-(methylthio)quinolin-3-yl)methanamine* S4**.

M-xylylenediamine (0.10 g, 0.74 mmol) was dissolved and stirred in 3 mL of *N,N-dimethylformamide* (DMF) under inert conditions. Potassium carbonate (0.20 g, 1.48 mmol) was then added to the solution. In a separate round bottom flask, 2-(methylthio)quinoline-3-carbaldehyde (0.15 g, 0.74 mmol) was dissolved in 3 mL of DMF, and this solution was added to the m-xylylenediamine solution. The reaction solution was then heated to 50 °C and stirred for 22 hours. $NaBH_4$ (0.07 g, 1.85 mmol) was added, and the reaction solution was allowed to stir for an additional 1.5 hours at room temperature. The reaction solution was quenched with the addition of DI H_2O (25 mL). The reaction was extracted with dichloromethane (2x 10 mL). The organic layers were then combined, washed with saturated aqueous NaCl, dried over upon the addition of 5 mL of brine. Combined organic layers were dried over Na_2SO_4 and concentrated under reduced pressure. The crude mixture was purified by flash column chromatography (SiO_2, 1 × 10 cm, 0−60% CH_3OH/dichloromethane gradient elution) to provide **S4** (27 mg, 12%) as a yellow solid. ^{1}HNMR (CD_3OD, 400 MHz): δ 8.07 (s, 1H), 7.91 (d, *J* = 8.4 Hz, 1H), 7.82 (d, *J* = 7.2 Hz, 1H), 7.65 (t, *J* = 7.2 Hz, 1H), 7.48-7.36 (m, 5H), 4.05 (s, 2H), 3.89 (q, *J1* = 8.4 Hz, *J2* = 4.4 Hz, 4H), 2.69 (s, 3H). ^{13}CNMR (CD_3OD, 100 MHz): δ160.62, 148.79, 141.82, 136.46, 135.37, 132.07, 130.72, 130.54, 130.30, 130.16, 128.97, 128.88, 128.69, 127.45, 126.77, 54.03, 50.06, 44.92, 13.27. IR (film) vmax 3376, 2924, 2853, 1646, 1599, 1559, 1490, 1457, 1396, 1330, 1313, 1136, 1046, 782, 753, 700 cm^{-1}. HRMS (ESI) *m/z* [M+H]+: calcd for $C_{19}H_{22}N_3S$ 324.1529; found: 324.15209.

Synthesis of S5 and S6

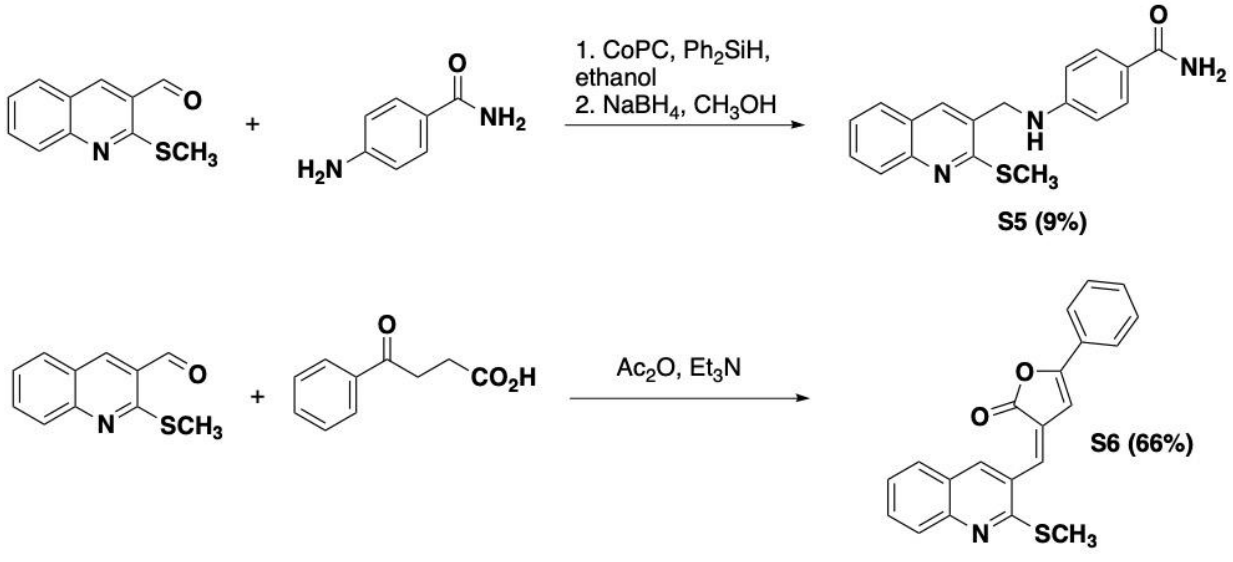

Scheme S2. Synthesis of compounds S5-S6.

4-(((2-(methylthio)quinolin-3-yl)methyl)amino)benzamide (S5)

Using the procedure from Kumar *et al.*,[1f] To a stirred suspension of cobalt phthalocyanine (CoPc, 4mg, 7.3 umol) in ethanol (3.65 mL) were added 2-(methylthio)quinoline-3 carbaldehyde (150 mg, 0.73 mmol), 4-aminobenzamide (99 mg, 0.73 mmol) and diphenylsilane (0.2mL, 1.1 mmol) at room temperature. The reaction was then warmed to 70 °C and allowed to stir for 20 h. Upon completion the reaction was filtered through Na_2SO_4 and concentrated under reduced pressure. The crude mixture was then purified via flash column chromatography (SiO_2, 3 × 15 cm, 45% ethyl acetate/hexane) providing the imine as a yellow solid. The imine was then diluted in anhydrous methanol (0.5 mL) under inert conditions and $NaBH_4$ (4 mg, 0.084 mmol) which provided desired amine **S5** as a pale yellow solid (20 mg, 9%). ^{1}HNMR (DMSO-d6, 400 MHz): δ 8.01 (s, 1H), 7.90 (d, *J* = 8.4 Hz, 1H), 7.85 (d, *J* = 7.2 Hz, 1H), 7.70-7.63 (m, 3H), 7.57 (br, 1H, NH), 7.46 (t, *J* = 8.0 Hz, 1H), 6.91-6.88 (m, 2H, NH), 6.56, (d, *J* = 9.2 Hz, 2H), 4.39 (d, *J* = 5.6 Hz, 2H), 2.70 (s, 3H). ^{13}CNMR (DMSO-d6, 100 MHz): δ 167.91, 158.34, 150.74, 146.51, 132.58, 130.28, 129.16, 127.77, 127.16, 125.53, 125.43, 121.73, 111.06, 42.93, 12.45. IR (film) vmax 3336, 3156, 1643, 1604, 1573, 1524, 1392, 1328, 1058, 804 cm^{-1}. HRMS (ESI) *m/z* $[M+H]^+$: calcd for $C_{18}H_{18}N_3OS$ 324.1165; found: 324.11567.

3-[[2-(Methylthio)-3-quinolinyl]methylene]-5-phenyl-2(3H)-furanone (S6)

Using the procedure from Abdelbaset *et al.*,[2] 2-(methylthio)quinoline-3 carbaldehyde (50 mg, 0.249 mmol) and 3-benzoylpropionic acid (47 mg, 0.27 mmol) were reacted with acetic anhydride and triethyl amine to produce **S6** (18.1 mg, 66% yield) as a red clay colored powder after recrystalization in dichloromethane. ^{1}HNMR (DMSO-d6, 400 MHz): δ 8.68 (s, 1H), 8.12 (d, *J* = 8.4 Hz, 1H), 7.94-7.91 (m, 3H), 7.82-7.78 (m, 1H), 7.69 (s, 1H), 7.62-7.54 (m, 4H), 7.50 (s, 1H), 2.71 (s, 3H). ^{13}CNMR (DMSO-d6, 100 MHz): δ 168.4, 159.5, 157.8, 147.6, 136.4, 131.9, 131.5, 129.6, 129.5, 128.2, 127.8, 127.7, 126.9, 126.5, 126.0, 125.7, 101.5, 13.30. IR (film) vmax 2186, 2119, 2109, 2099, 2081, 2032, 1997, 1773, 1582, 1546, 1490, 1447, 1395, 1360, 1315, 1247, 1241, 1167, 1144, 1066, 1048, 998, 920, 881, 815, 779, 758, 738, 685 cm^{-1}. HRMS (ESI) *m/z* [M+H]+: calcd for $C_{21}H_{16}NO_2S$ 346.0896; found: 346.08890.

Initial antibacterial activity screen of compounds 4, 5, and S1-S6

Compounds **4**, **5**, and **S1**-**S6** were screened for initial antibiotic activity against a susceptible laboratory strain of EC (designated EC 25922), and non-virulent, biofilm forming strain of PA (designated PA 9027), and PΔ6 (efflux knockout strain[6]) at 128 μg/mL in DMSO. Sterilization and Antimicrobial Susceptibility Assay procedures utilized were the same as described in the methods.

Table S1. Antibacterial activity screening results for compounds 4, 5, and S1-S6 at 128 μg/mL

Table S1. Antibacterial activity screening results for compounds 4, 5, and S1-S6 at 128 μg/mL.			
**Compound**	**EC 25922**	**PA 9027**	**PΔ6**
**4**	NI	NI	I
**5**	I	NI	I
**S1**	NI	NI	NI
**S2**	NI	NI	NI
**S3**	NI	NI	NI
**S4**	NI	NI	NI
**S5**	NI	NI	NI
**S6**	NI	NI	NI
I = inhibition of cell growth at 128 ug/mL, NI = no inhibition of cell growth at 128 μg/mL.			

NMR Spectra

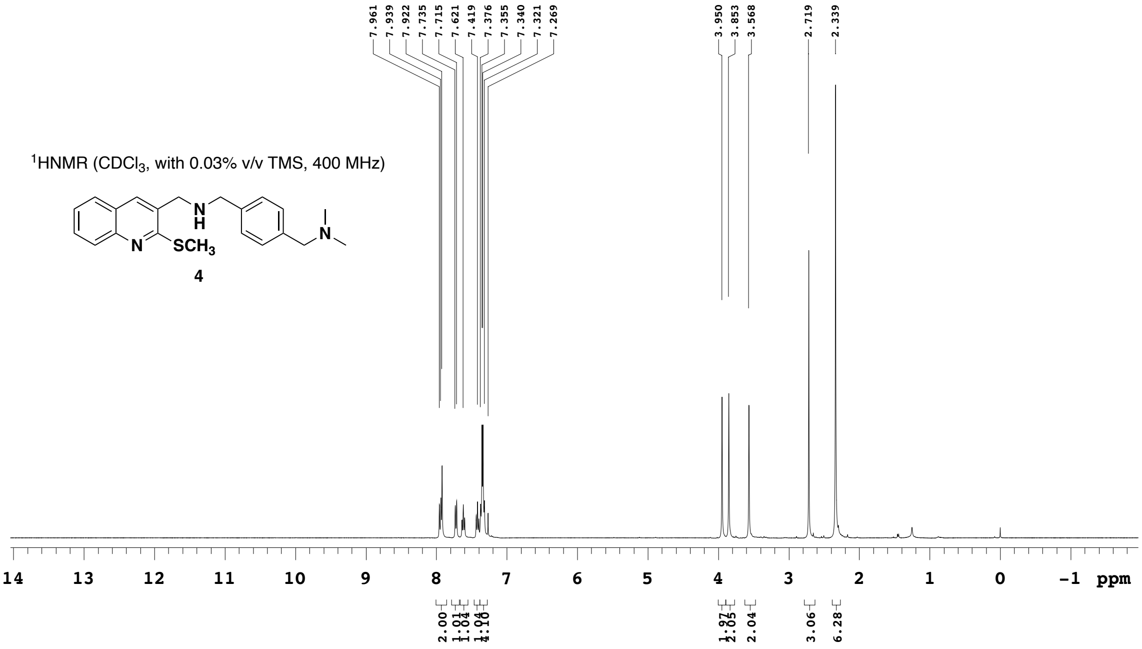

7.961
7.939
7.922
7.735
7.715
7.621
7.419
7.376
7.355
7.340
7.321
7.269
3.950
3.853
3.568
2.719
2.339
^{1}HNMR ($CDCl_3$, with 0.03% v/v TMS, 400 MHz)
N
H
N
SCH3
N
4
14 13 12 11 10 9 8 7 6 5 4 3 2 1 0 -1 ppm
2.00
1.01
3.06
6.28
2.04

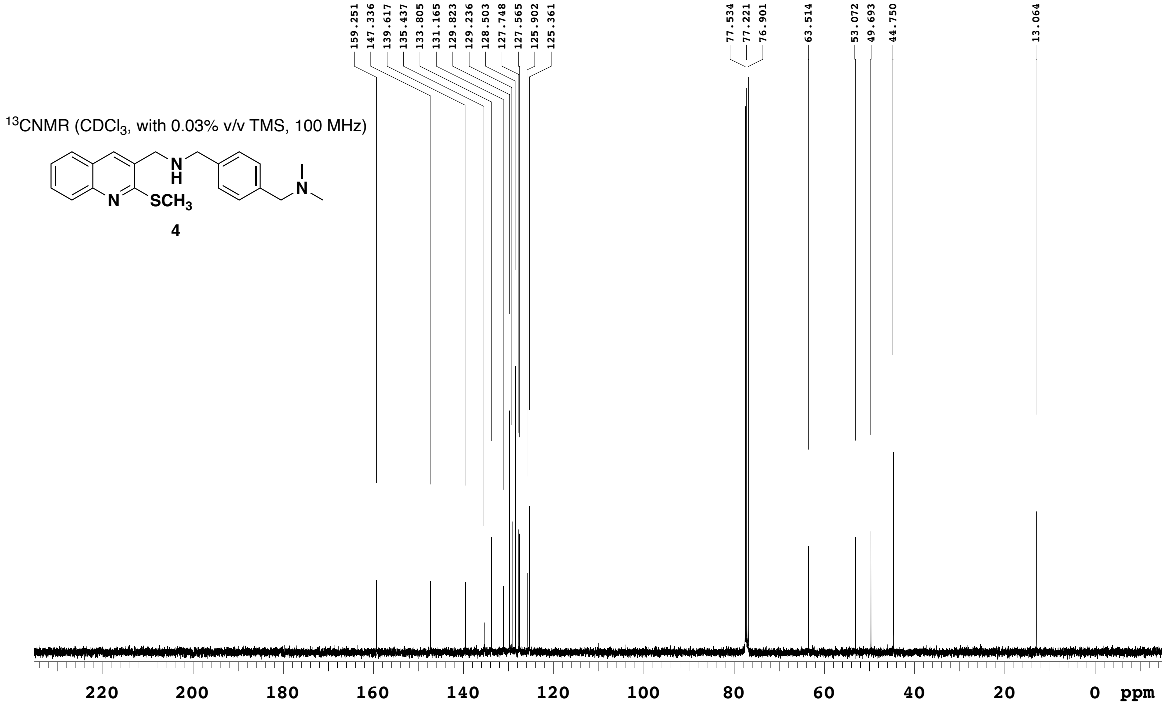

159.251
147.336
139.617
135.437
133.805
131.165
129.823
129.236
128.503
127.748
127.565
125.902
125.361
77.534
77.221
76.901
63.514
53.072
49.693
44.750
13.064
^{13}CNMR ($CDCl_3$, with 0.03% v/v TMS, 100 MHz)
N
H
N
SCH3
N
4
220 200 180 160 140 120 100 80 60 40 20 0 ppm

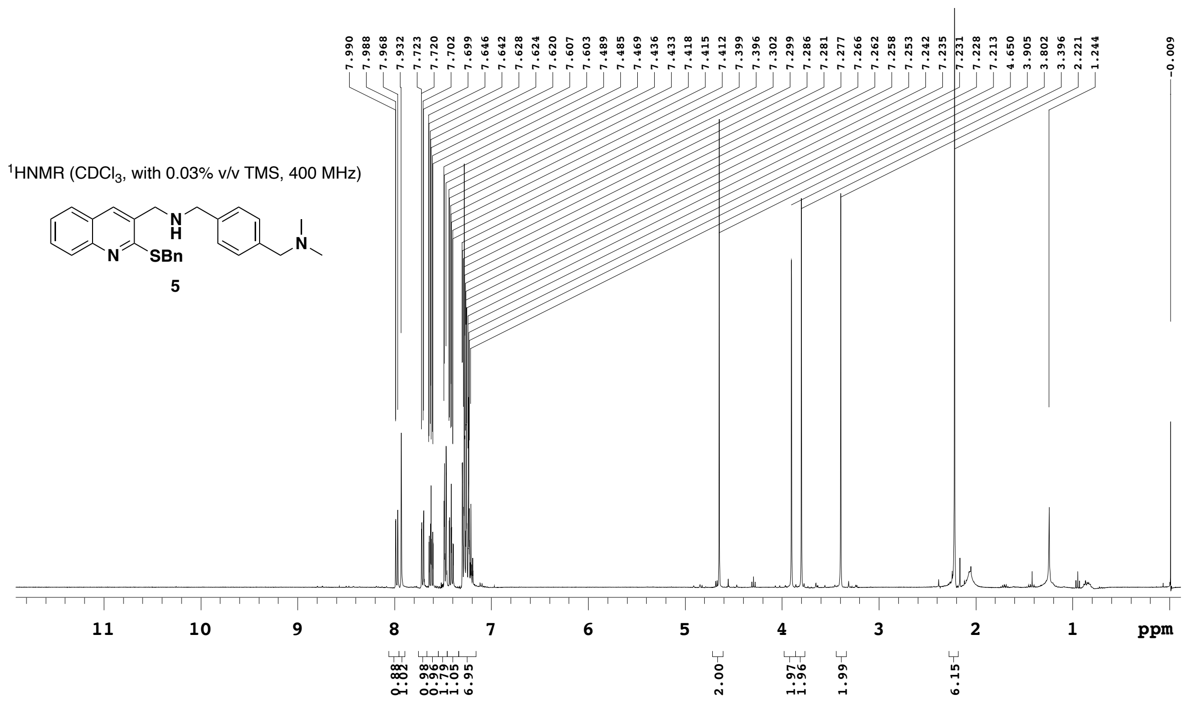

^{1}HNMR (CDCl$_3$, with 0.03% v/v TMS, 400 MHz)
N
H
N
SBn
N
5
7.990 7.988 7.968 7.932 7.723 7.720 7.702 7.699 7.646 7.642 7.628 7.624 7.620 7.607 7.603 7.489 7.485 7.469 7.436 7.433 7.418 7.415 7.412 7.399 7.396 7.302 7.299 7.286 7.281 7.277 7.266 7.262 7.258 7.253 7.242 7.235 7.231 7.228 7.213 4.650 3.905 3.802 3.396 2.221 1.244 -0.009
11 10 9 8 7 6 5 4 3 2 1 ppm
0.88 1.02 0.98 0.96 1.79 1.05 6.95 2.00 1.97 1.96 1.99 6.15

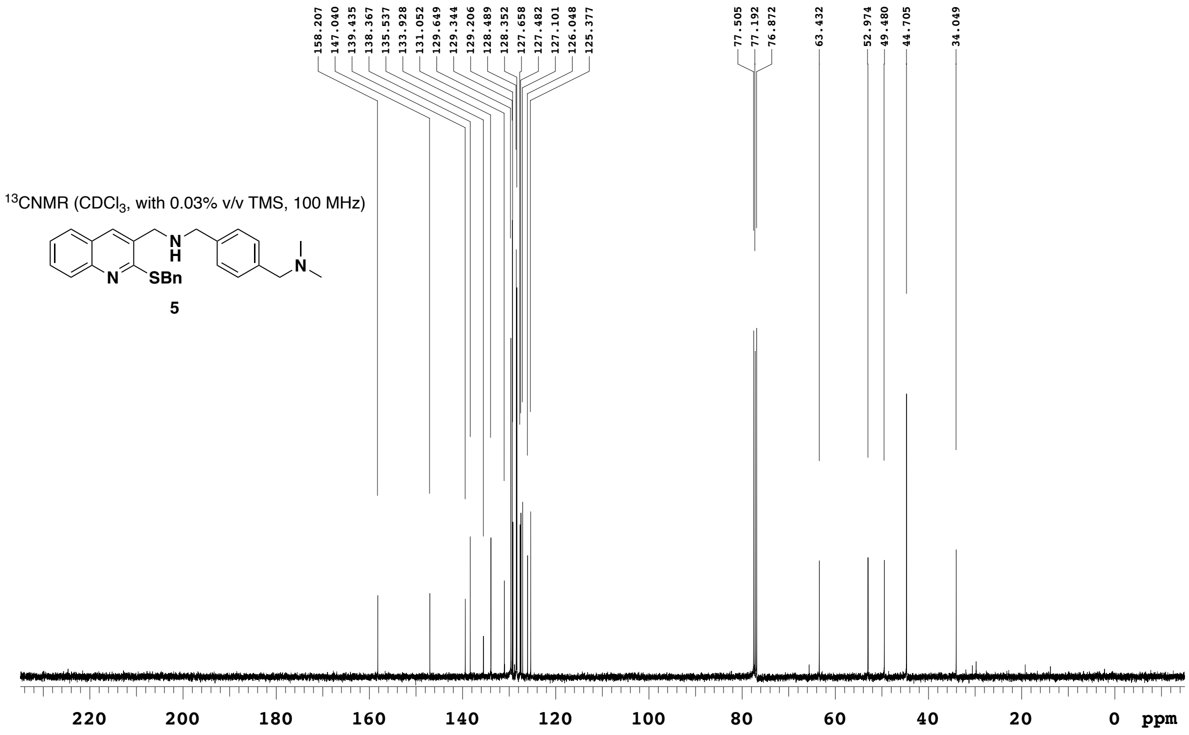

^{13}CNMR (CDCl$_3$, with 0.03% v/v TMS, 100 MHz)
N
H
N
SBn
N
5
158.207 147.040 139.435 138.367 135.537 133.928 131.052 129.649 129.344 129.206 128.489 128.352 127.658 127.482 127.101 126.048 125.377 77.505 77.192 76.872 63.432 52.974 49.480 44.705 34.049
220 200 180 160 140 120 100 80 60 40 20 0 ppm

PA ATP Synthase Primers for PCR

Primers were developed using the *atp* operon from PA01 DNA genome sequence, which is the most similar to ATCC 9027.[3] Forward and reverse primers introduced NdeI and HindIII restriction sites (highlighted), respectively:

Forward: 5'-CTGGACTACCATATGTGGTGGTCACAGGCAAAACCTGTGA-3'
Reverse: 5'-GATCAAGCTTCTGATTCTGACGGCGGTGCTCTTCG-3'.

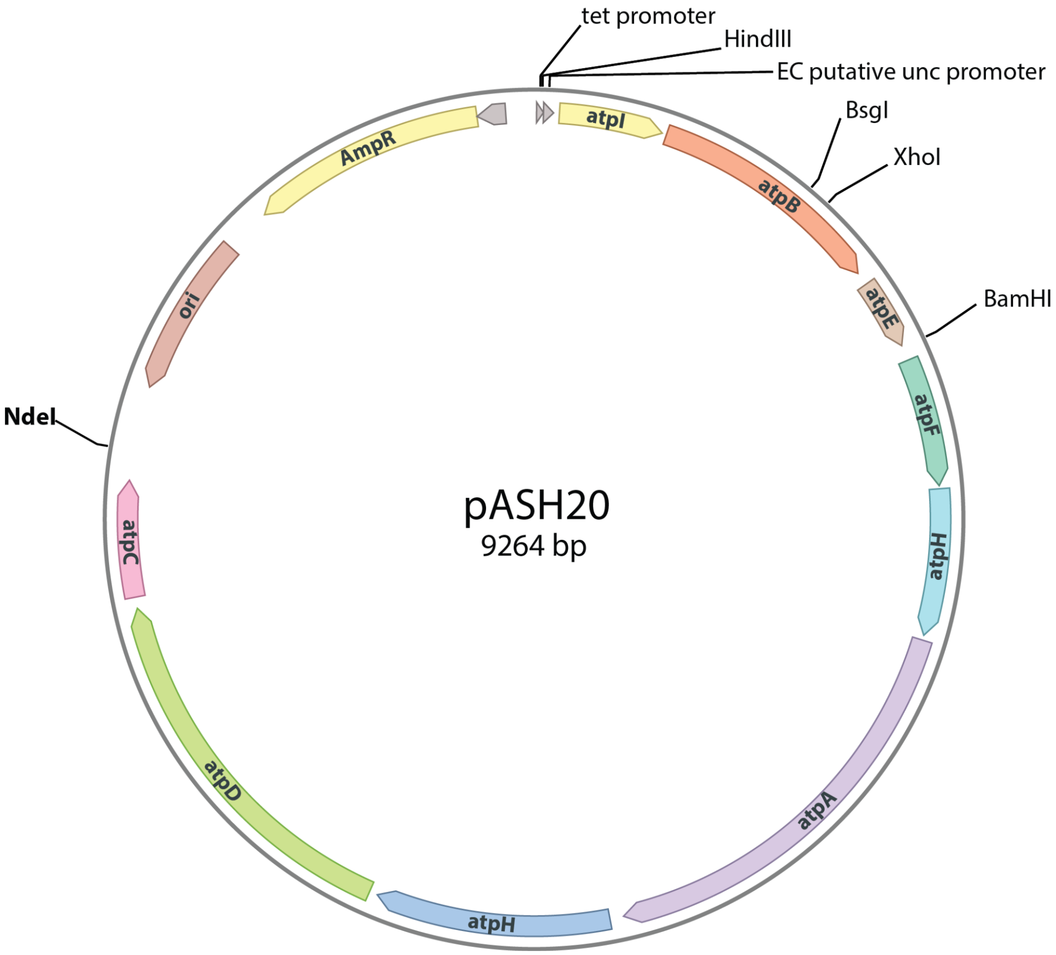

Figure S1. Map of pASH20 plasmid showing relevant restriction sites and gene annotations.

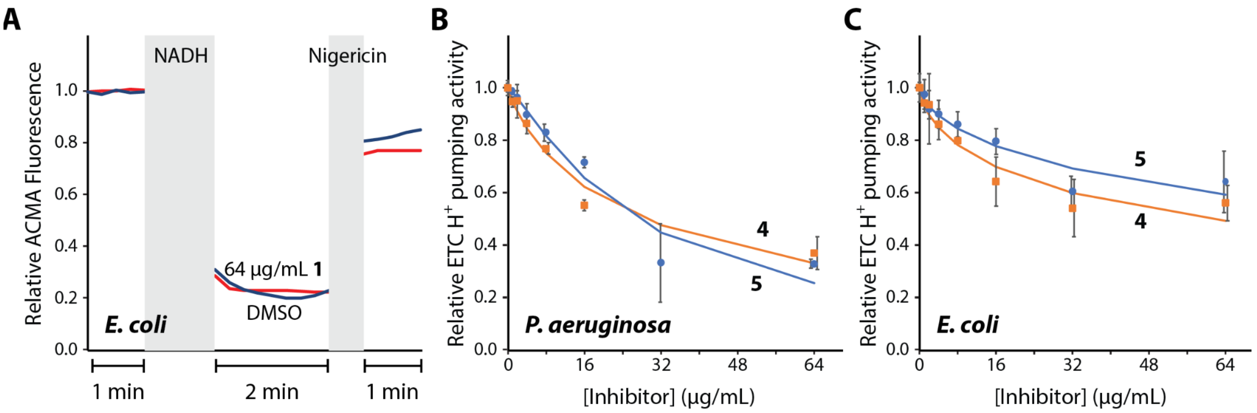

Figure S2. *Inhibition of ETC-driven H$^+$ pumping does not account for inhibition of ATP synthesis activity*. H$^+$ pumping in inverted membrane vesicles from EC or PA strains was measured using ACMA dye as described below (*Supplemental Methods*). **A**) Compound **1** did not inhibit ETC activity in this assay even at 64 µg/mL, as indicated by the quenching of ACMA fluorescence after addition of NADH. Compounds **4** and **5** showed some inhibition of the PA ETC (**B**) and mildly inhibited the EC ETC (**C**).

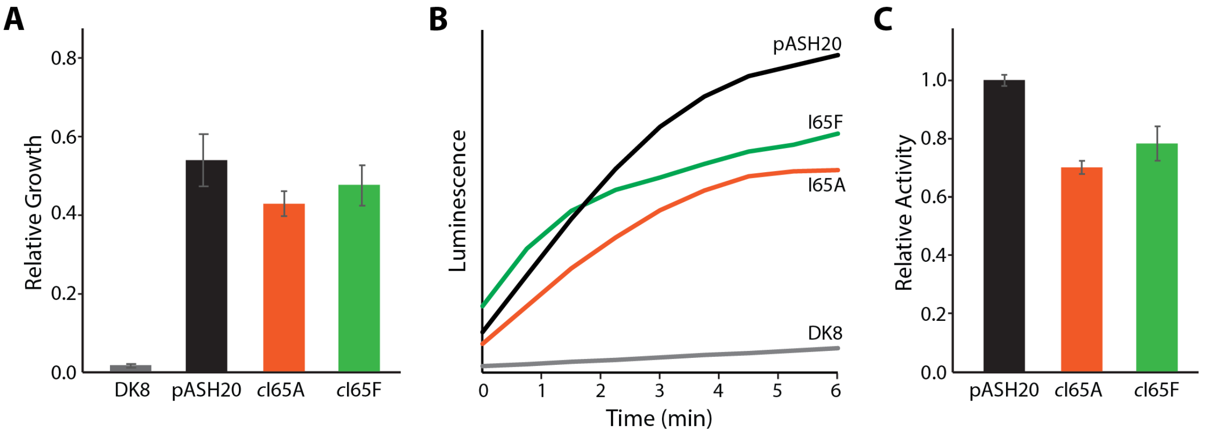

Figure S3. *Mutation of cIle65 to Ala or Phe does not significantly inhibit ATP synthesis activity*. **A)** *E. coli* DK8 cells transformed with pASH20 (wildtype) or cIle65 mutations were grown in succinate minimal medium as described in *Methods*. Bars show average and standard deviation of n≥3 replicates relative to the growth of DK8 pFV2. **B)** Inverted membrane vesicles prepared from the *E. coli* strains in **A** were assayed for ATP synthesis activity using the continuous luminescence assay described in *Methods*. **C)** ATP synthesis activity with no inhibitor was determined using the endpoint ATP synthesis assay as described in *Methods*. Background luminescence (in the presence of CCCP) was subtracted, and resulting luminescence was normalized to that of pASH20 to show relative effect of the mutations on activity.

Table S2: All IC_{50} values and Hill coefficients for dose response fits of inhibition of ATP synthesis activity.

	IC_{50} in µg/mL (Hill coefficient)		
**Vesicles**	**1**	**4**	**5**
PA	10.0 (1.4)	11.1 (0.8)	0.7 (0.8)
DK8/pASH20	10.6 (1.3)	30.3 (1.0)	2.3 (1.3)
DK8/pASH20 *c*I65A	6.9 (1.1)	20.9 (1.2)	5.7 (1.7)
DK8/pASH20 *c*I65F	8.4 (1.4)	4.5 (1.4)	3.5 (1.8)

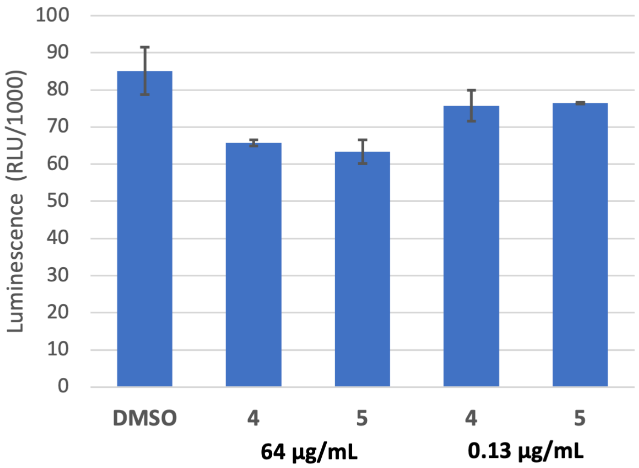

Figure S4. *Compounds 4 and 5 did not significantly inhibit luciferase after 500-fold dilution*. Compounds (or an equivalent volume of DMSO) were mixed with luciferase assay solution (50 mM Tricine-NaOH, pH 7.8, 10 mM $MgSO_4$, 0.2 mM EDTA, 0.2 mM NaN_3, 1 mM dithiothreitol, 150 µg/mL luciferin, and 7.5 µg/mL luciferase) at the indicated concentrations, and luminescence was measured after the addition of ATP to 0.2 µM. Bars show the average and standard deviation of three replicates.

Supplemental Methods

Determination of electron transport activity. In an opaque black 96-well plate, inverted membrane vesicles from PA or *E. coli* DK8 pASH20 were added to HMK buffer containing 0.3 µg/mL 9-amino-6-chloro-2-methoxyacridine (ACMA) and 0-64 µg/mL test compound in DMSO. After measurement of baseline ACMA fluorescence for 60 s, redox-driven H^+ pumping was initiated by addition of NADH to 0.8 mM, and fluorescence quenching was monitored for 120 s before addition of nigericin to 0.5 µg/mL. Relative activity is reported as the minimum fluorescence value normalized to the maximum fluorescence value following addition of nigericin.

Supplemental References

1. Kumar, V., Sharma, U., Verma, P. K., Kumar, N. and Singh, B. (2012), Cobalt(II) Phthalocyanine-Catalyzed Highly Chemoselective Reductive Amination of Carbonyl Compounds in a Green Solvent. *Adv. Synth. Catal.* 354, 870-878. DOI: 10.1002/adsc.201100645
2. Abdelbaset, M. S., Abuo-Rahma, G. E-D. A., Abdelrahman, M. H., Ramadan, M., Youssif, B. G. M., Bukhari, S. N. A., Mohamed, M. F. A., Abdel-Aziz, M. (2018) Novel pyrrol-2(3H)-ones and pyridazin-3(2H)-ones carrying quinoline scaffold as anti-proliferative tubulin polymerization inhibitors. *Bioorganic Chemistry* 80, 151-163. DOI: 10.1016/j.bioorg.2018.06.003.
3. Winsor, G. L.; Griffiths, E. J.; Lo, R.; Dhillon, B. K.; Shay, J. A.; Brinkman, F. S. L. (2015) Enhanced annotations and features for comparing thousands of *pseudomonas* genomes in the pseudomonas genome database. *Nucleic Acids Research* 44(D1), D646–D653 DOI: 10.1093/nar/gkv1227.
